# Presidential Address: Tropical Medicine in War and Peace†

**DOI:** 10.4269/ajtmh.15-0836

**Published:** 2016-07-06

**Authors:** Christopher V. Plowe

**Affiliations:** ^1^ Institute for Global Health, University of Maryland School of Medicine, Baltimore, Maryland.

Each of us—each of you—sitting here tonight is working hard every day to make the world a better—and a healthier—place. Whether you dispense pretravel advice and vaccines or splice trypanosome genes, whether you are responding to the Ebola epidemic or designing a malaria-refractory mosquito, the spirit that animates your work is one of beneficence, generosity, and peace.

But we do not live in a beneficent and peaceful world.

In 1903, the year our parent society, the American Society of Tropical Medicine, was formed, 15 wars were taking place worldwide. Most of these conflicts were between an imperial power—like the British Empire or Ottoman Empire or the United States—and much smaller, less powerful independence movements or indigenous peoples—Native American tribes like the Apache and Cheyenne or the Moro people of the Philippines.

In these wars, the big guy usually wins—although I was interested to discover that Brazil seems to have handed a defeat to the United States and Bolivia in 1903. I also learned that even 112 years ago, conflict in the Middle East foreshadowed today's chronic violence there. Several of these wars were fought over commercial interests—both the Acre War on the Brazil–Bolivia border and Bailundo Revolt in Angola involved rubber plantation workers who revolted against forced labor, or whose livelihoods were threatened by economic or political changes. Life has not gotten much better for many plantation workers in the last 100 years—earlier this year mass graves containing the bodies of dozens of Burmese migrant workers were found in forced labor camps on rubber plantations on the Thailand–Malaysia border.

Tonight, as we sit here in Philadelphia at the 64th annual meeting of the American Society of Tropical Medicine and Hygiene (ASTMH), no fewer than 54 wars are being waged worldwide—defining war as the use of armed force between two or more organized armed groups resulting in at least 100 deaths. Major wars—those resulting in more than 10,000 deaths in the last year—include the conflicts in Syria, Iraq, and Afghanistan and the Boko Haram insurgency in West Africa. Thousands have also died in the last year in each of another 14 conflicts in the Middle East, Asia, Africa, and one in Europe, the war in Ukraine, which has resulted in nearly 8,000 deaths since last year.

I do not know about you, but sometimes I find myself exhausted by the constant drumbeat of grim news about wars, brutality, and suffering. As scientists and health professionals, how do we see ourselves and our work in relation to war and violence? Do we try to just set aside these disturbing images and continue to do our small part to make the world a little better, putting one foot in front of the other each day and hoping that our collective efforts will someday, somehow win out against the forces of death and destruction?

Maybe as individuals we do—and maybe we should—sometimes turn our attention away from all the bad news and focus on the good we can do through our work in our communities and in our families. But as tropical medicine and global health professionals—as the ASTMH—we also have a long history of responding to the demands of wartime by making discoveries, developing new treatments, and creating new institutions that ultimately improve global health in important and lasting ways.

The connection between wars and tropical medicine is obvious when you look at the world map, whether of malaria or of neglected tropical diseases—there is simply more war in the tropical, less economically developed countries and more disease in war zones and conflict areas.

This evening I will describe a few well-known examples of how we have transformed the destructive forces of war between humans into new weapons in the war between humans and the tropical disease agents—the viruses, parasites, and other bugs—that we all do battle with and hope eventually to conquer. And then I will tell you about some of my own recent experiences that have led me to think that maybe we can turn this equation around—examples of how our work in tropical medicine and global health can serve as a way to promote peace.

Tonight will not be the first time that an ASTMH president has talked about the need for our Society to step outside our comfort zones in the laboratory, in the clinic, and in the field and get actively engaged in politics and advocacy. In his presidential address at the 1992 ASTMH meeting in Seattle, WA, Don Krogstad told us that “… health has a universal appeal that transcends politics, and is therefore a sound long-term investment in foreign relations that does not become outdated when political power changes hands.”

In Baltimore, MD, in 1996, Don Burke called on us to become activists for tropical medicine and hygiene, telling us he believed “… that this Society can be an important voice in a chorus calling out for international cooperation and common purpose to address global health issues.” And in Denver, CO, in 2002, Michele Barry talked about globalization and again challenged us to become activists, arguing that ASTMH should be “… more of a public advocate for tackling the global health disparities that have widened dramatically during the era of globalization.”

And just 4 years ago here in Philadelphia, PA, Peter Hotez introduced the idea that tropical disease elimination can serve as a means to implement international science diplomacy. Peter pointed out both the effects of tropical infections on promoting war and conflict and the opportunity that shared suffering from tropical diseases provides for us to bring people together to achieve health goals.

Tonight I want to take a look at things from a slightly different perspective. In addition to looking at how disease promotes conflict or how common suffering promotes cooperation on health goals, let us think about whether there is a potential for commonly shared public health aims to foster political and social reconciliation—let us ask if tropical medicine be a catalyst for peace?

Something that Don Burke told us in his talk 19 years ago struck me as true then, but possibly not so much still the case in today's much more globalized and connected world. Don analyzed how ASTMH membership rises and falls in relation to wars, and he concluded that “Membership in our Society predictably surges during conflicts where the U.S. national interests are perceived to be directly threatened by tropical diseases, then stagnates in the inevitable post-war national doldrums. The patterns are clear. It should come as no surprise that International Politics drives tropical medicine, and not the reverse.”

This indeed was the pattern during our Society's first century—our membership surged when tropical diseases like malaria and typhoid fever caused heavy troop casualties in World War II and Vietnam. Our leading public health and research institutions likewise were created in response to and shaped by our war efforts. The Malaria Control in War Areas (MCWA) was established in 1942 to combat malaria around military training bases within the United States, and after the war, the MCWA became the Centers for Disease Control and Prevention (CDC), which at first stood for “Communicable Disease Center.”

The National Institutes of Health (NIH) creation story goes even further back, to 1887, when a one-room laboratory was set up at the Marine Hospital on Staten Island to monitor returning merchant seamen and prevent epidemics of cholera and yellow fever. In 1912, the Marine Hospital Service became the Public Health Service, and during World War I, the Public Health Service's Hygienic Laboratory investigated anthrax outbreaks among the troops and was able to put the blame on contaminated shaving brushes. That small laboratory became the National Institute of Health—with no “s” after Institute—in 1930, and NIH expanded dramatically during World War II, in large part to develop vaccines for tropical diseases like typhus and yellow fever that were causing heavy troop casualties, and especially to make new synthetic antimalarial drugs.

In parallel with the race to build a nuclear bomb, German and American scientists were also in a race during the World War II to find a new drug for malaria, which at that time could only be treated with one drug, quinine, which had to be extracted from the bark of the cinchona tree. Many thousands of Allied soldiers died of malaria in the African and Pacific theaters after the Japanese took control of the world's supply of quinine by occupying Indonesia, where quinine was produced on cinchona plantations on the island of Java.

Another pharmaceutical arms race took place during the Vietnam War. North Vietnamese troops started dying in large numbers along the Ho Chi Minh trail, not from American bombing but from chloroquine-resistant falciparum malaria. At around the same time that the U.S. Army embarked on a drug development program that eventually produced mefloquine, the North Vietnamese turned to China for help. This was during Chairman Mao's Cultural Revolution, when most intellectuals, including scientists, were being shipped off to reeducation camps.

But Chinese malariologists like Tu Youyou and her colleagues were spared from the camps and tasked with finding a replacement for chloroquine. Combing through ancient records of traditional herbal treatments they came across the plant qinghausu, which had been used for at least 2,000 years to treat intermittent fevers. In a work that was recognized a few weeks ago by a Nobel Prize, Dr. Tu and her colleagues carefully read those old texts and worked out how to extract the active compounds, the artemisinins, and they and others developed this lifesaving class of antimalarial drug that is used worldwide today in the form of artemisinin-based combination therapy.

If you want more evidence of the connection between military action and advances in tropical medicine and global health, you can turn and look at the men and women sitting next to you. So many of the brightest lights in tropical medicine, from Ronald Ross and Walter Reed to Alan Magill, wore a military uniform and, like Alan did, served during wartime. I will never forget Alan describing to me how, when he saw the massive invasion force of war machines rumbling across the desert toward Baghdad, he thought about how much good we could do if even a fraction of the resources on display were deployed to fight tropical diseases.

So I think we can agree that while war of course has absolutely devastating negative consequences for global health, there is also a sort of perverse calculus in which we might say that war has been good for global health and tropical medicine, in the sense that war and conflict have indirectly led, again and again, to lifesaving new drugs and vaccines and institutions that ultimately might end up saving many more lives than those lost in wars.

As Don Burke observed, during the twentieth century our numbers in ASTMH—and our funding in tropical medicine—have indeed grown during wartime and dwindled during peacetime. But I am not sure that Don's observation that “International Politics drives tropical medicine, and not the reverse,” is still true today, or that it still has to be true in any case. I believe—and I hope to convince you—that in the twenty-first century, tropical medicine and global health can become forces for political reconciliation and peace.

In thinking about how we as individuals and as a Society respond to violence and war, I go back to my own childhood in South Dakota. My father was an Episcopal priest ministering to the Lakota Sioux Indian people on the Pine Ridge and Rosebud Reservations, which encompass South Dakota's famous Badlands. The reservation has always been a violent place. In 1890, just 40 miles from the little town of Martin, South Dakota, where I grew up, the U.S. Seventh Cavalry Regiment massacred 200 Lakota Sioux, mostly old men, women, and children at Wounded Knee on the Pine Ridge Reservation.

When I was in junior high school, a group of 200 Lakota Sioux occupied the same town of Wounded Knee to protest corruption and abuse. The U.S. Marshals and Federal Bureau of Investigation moved in, the shooting started, and there were deaths on both sides before tribal elders called an end to the protest after more than 2 months of fighting. So this was the background of my childhood, and I personally experienced violence on a more than one occasion, including getting badly beaten up at an age of 13 years by four older high school boys. My response to getting beaten down and kicked in the face was to carry a great big folding knife in my back pocket everywhere I went for the next several years—including to school every day, if you can imagine that—it was a different time.

And that is a natural response to violence—to get a weapon, to learn karate, to form a militia—to fight. I know someone else who had the same response to violence, initially, at least. In the summer of 1988, medical students at Rangoon University in Myanmar, the southeast Asian country formerly known as Burma, were looking forward to their last year of medical school. These students' lives were shattered in August of that year by a violent uprising in Burma. Students—including medical students—were at the forefront of national protests against the ruling military regime. The students actually briefly seemed to carry the day, and they convinced a young academic, Aung San Suu Kyi, to be their leader.

But that heady moment did not last. The protests were brutally put down by the military. Peacefully protesting nurses and students were gunned down in the streets. Thousands were killed. Many more were imprisoned, and others went into exile. Large numbers of Burmese students congregated on the Burma–Thailand border, where they linked up with some of the ethnic armies that had been fighting the military government and seeking independence for decades. The students had a very understandable response to the violence that had been inflicted on them—they armed themselves and tried to raise an army to go back and take on the Burmese military.

The students' plan to fight did not get very far. The military government of Burma stayed in power and became increasingly isolated and xenophobic. Economic sanctions imposed by the United States and other western countries only increased the isolation. Most of the students eventually gave up their fight, scattered to the four winds, and tried to rebuild their lives. One young medical student who had joined the protest and then taken up arms on the border lived briefly in Thailand as an illegal immigrant, then sought asylum in England before coming to the United States where she started her medical training all over again, completing premedical studies, medical school, clinical training, and a PhD, eventually becoming a malariologist.

For many years, this former student activist was unable to go home to Burma—now called Myanmar. She was blacklisted by the government and would have gone from the airport straight to prison. She remained bitterly opposed to any sort of engagement with the Myanmar government and fully supported the sanctions.

There was another major antigovernment uprising in Myanmar in 2007, and this time led not by students but by Buddhist monks, who are highly respected and revered in Burmese society. This time the whole world was watching, but despite the international attention, the government's response was the same—more guns, more violence, more deaths. Our former young student rebel was dismayed—she had always believed that if only the rest of the world, and especially the United States, had known what was happening in 1988 they would have intervened. After seeing the Saffron Revolution end the same way the '88 uprising did—this time, despite the whole world watching it unfold on the Internet and doing nothing to help—our former student rebel's thinking changed. Raising an army to fight back was futile, decades of sanctions did nothing.

Maybe it was the time to take a different approach.

By this time the onetime rebel—my wife, ASTMH member Myaing Myaing Nyunt—was married to, and working with, another malaria researcher. Together the two of us decided to reach out to our fellow malariologists inside Myanmar. Slowly, and quietly, in 2009 we began collaborating with both civilian and military government malaria researchers.

We chose to work with the Burmese military, despite their human rights record, for several reasons. First, many of the places in Myanmar with the most malaria are in conflict areas that only the military can reach. We cannot make progress against malaria in Myanmar without working with the military along with the Ministry of Health and the private sector. And we found that there were some very well-trained and dedicated doctors and military scientists working on malaria, who shared the motivation that we all have to improve the health of their countrymen.

Finally, by engaging the military medical corps, we hoped to begin building links between the military and other groups within Myanmar who have been working in isolation from each other. We were the first foreign visitors to the Defense Services Medical Research Center in the hills outside the capital, Nay Pyi Taw, in 2011. So now you have the former rebel, not only shaking hands with uniformed Burmese soldiers but also working with a brigadier general and his team of military medical officers to conduct molecular surveillance in support of malaria elimination.

Not long after we started working there, things started changing in Myanmar, for reasons that are not very clear. Aung San Suu Kyi was released from nearly two decades of house arrest in 2010, and the military government transferred power to a nominally civilian government in 2011, albeit one dominated by very recently retired military officers. In 2012, the United States resumed full diplomatic relations with Myanmar, and the next year both Secretary of State Clinton and President Obama visited and met with both President Thein Sein and opposition leader Aung San Suu Kyi. This thawing of relations made it possible for us to get the first-ever NIH grant to work inside Myanmar with Myanmar government scientists.

This grant gave us an opening to organize training in ethics for our collaborators, including the military. To receive U.S. federal funding for human subjects research, institutions have to have an ethical review committee—or institutional review board (IRB)—and that IRB has to apply for what is called a “Federalwide Assurance” from the Office of Human Research Protections at the Department of Health and Human Services, certifying that their IRB meets several criteria intended to assure protection of human research subjects—essentially a license to do human research.

One important way of building political will for malaria elimination, we believe, is to build local capacity for malaria research and surveillance. Part of Myaing's vision is also to use malaria research as a way to start conversations in Myanmar about things like ethics and professionalism—with support from the NIH Fogarty International Center, she organized training workshops in research ethics and helped both the civilian and military IRBs get their Federalwide Assurances.

Despite the importance of human rights issues in Myanmar, overtly using human rights as a framework for discussion with the Myanmar government at that delicate time was not realistic. But what is the protection of human subjects and informed consent, if not human rights?

And so we had the former rebel training the soldiers in ethics.

Myaing also convinced George Soros, through his Open Society Foundations, to support training in clinical ethics and professionalism for medical students. One of her projects is called “Malaria as a Catalyst for Social Change”—her idea was to use a shared goal of combating malaria to build new trust and cooperation between diverse groups in Myanmar, including not just civilian and military malaria workers but also community-based organizations associated with the ethnic militias in border areas who had long histories of conflict with the government.

Malaria affects everybody—soldiers and rebels, farmers and gold miners, and rubber plantation workers. Even if the government and border groups are unable to reach political agreement, surely, we hoped, they can agree to eliminate malaria. And maybe, just maybe, starting a conversation and getting agreement about shared health goals like malaria elimination could help establish the beginnings of trust and understanding where there was none before.

Of course there have been other examples of using shared health goals to foster peace, at least for a little while. In El Salvador, Sri Lanka, Afghanistan, and many other countries, warring parties have agreed to temporary cease-fires to allow people on both sides to be vaccinated against smallpox, polio, and childhood diseases. But these “Days of Tranquility” are only temporary——one day, the shooting stops so that the vaccination teams can do their work—and the next day the shooting starts again.

Can we use shared health goals to pursue more permanent peace?

As you know, our president last year, Alan Magill, brought Bill Gates to ASTMH as our keynote speaker. We had also invited the Myanmar National Malaria Control Program manager and the director of the equivalent of the NIH in Myanmar. They attended the ASTMH Council meeting last year, where they got to meet Bill and Alan. Later during the meeting, Alan sat down with our Myanmar colleagues and made a suggestion “Even though Myanmar has the most malaria of any country in Southeast Asia and many challenges to overcome, was it possible that Myanmar could make a decision to be not the last, but the first country in the region to eliminate malaria?”

Shortly before the ASTMH meeting last year, Alan came to Myanmar for the first time and came away very impressed by the quality and commitment of diverse members of the malaria community, not only from the government but also from the private sector and nongovernmental organizations (NGOs). Most of these organizations had limited interaction with the Ministry of Health and none at all with the military, but Alan could see the potential for cross-sectoral cooperation as Myanmar began to open up. Alan's specific suggestion to our Myanmar visitors last year was “Could malaria elimination serve as a national reconciliation campaign in Myanmar, much like Nelson Mandela made winning the World Cup a national goal to help reconcile post-apartheid South Africa?”

The next month, we brought together civilian and military, public and private partners to plan surveillance for malaria elimination. Now we had a leading Ministry of Health malaria expert, the country director of an NGO that worked with ethnic groups on the border that have long been in conflict with the government, the brigadier general, and our former rebel, now malariologist, working and eating together and sharing stories and jokes. Just 2 years earlier, this scene would have been inconceivable.

Some of the results of this malaria mapping project were presented 2 days ago at a symposium including partners from the Ministry of Health, the Ministry of Defense, and an NGO that works in ethnic border areas. Another partner is the Chinese CDC, whose workers are uniquely able to reach malaria-affected populations in Kachin and Shan states along the China–Myanmar border that are out of reach to government malaria workers inside Myanmar.

Just this last August 2015, we took this malaria diplomacy approach to another level. Together, ASTMH, the University of Maryland's new Institute for Global Health, and the Center for Strategic and International Studies (CSIS), a bipartisan think tank in Washington, organized a conference on malaria elimination in Myanmar. The goal was to bring together a diverse group of partners from Myanmar, many of whom had a long history of adversarial relationships and who had never sat down together before to talk about anything, and try to get them to agree to work together to eliminate malaria from Myanmar.

We were told that it would never work—that the opposition politicians would never agree to sit down with the government, that the ethnic health organizations would have nothing to do with the military, and that the State Department would never approve visas for high-ranking Myanmar military officers to travel to the United States.

I am happy to report that the event did happen and that it was a success. The Deputy Minister of Health, the Senior Health Advisor to the President of Myanmar, members of parliament from both the government party and the main opposition party, Aung San Suu Kyi's National League for Democracy, Suu Kyi's senior political advisor, an official from the Ministry of Foreign Affairs, two brigadier generals from the Myanmar Defense Medical Services, and four leaders of ethnic health organizations from conflict areas on the border, all showed up in Washington, DC, for this conference and were joined by leaders from the U.S. President's Malaria Initiative, the Gates Foundation, World Bank, the Asia Pacific Leaders Malaria Alliance, and the U.S. Military. The World Health Organization and the Global Fund to Fight AIDS, Tuberculosis and Malaria sent statements of support.

I will admit, it was a little tense at first. But over the course of 2 days, we saw new understanding and trust begin to blossom between these former adversaries, and by the end of the meeting, we heard very open and frank exchanges in this closed door meeting and saw an amazing coming together of all partners to agree to work together on a national malaria elimination campaign. There was a unanimous consensus that malaria elimination is too important, and too urgent, to wait for political developments like a fully free and fair election or a national cease-fire—malaria elimination should proceed no matter which way the political winds are blowing, whether in Myanmar or in the United States. The meeting was widely covered by the media both here and in Myanmar and the region, and a costed National Strategic Plan for malaria elimination is being drafted in an inclusive process even as politics marches on.

Can we credit the consensus on malaria elimination for a cease-fire or for a successful transfer of power in national elections in November 2015? Of course not—but I do think we can say with some confidence that tropical medicine and global health have had a meaningful, positive impact on political relationships in this emerging democracy. Health is an area where adversarial partners can agree when they can agree on little else, and it gives everybody an opportunity to take shared credit for doing something good for their people.

As the leading professional scientific organization in the world for tropical medicine and global health, I believe that ASTMH is uniquely positioned to leverage the scientific and public health diplomacy that is already a part of what we do every day and amplify it for greater impact beyond the laboratory and the clinic to benefit the broader societies. We had another example of how we can use our voice in the symposium held at the 2015 ASTMH annual meeting on building bridges between the United States and Cuba.

Our name is the “American” Society, and while we recognize the leadership that Americans took to establish ASTMH, we also recognize that we are now a very international society. As a result of some recent steps making it easier for our low- and low-to-middle income colleagues to join, our membership is now about one-third from outside the United States. We anticipate that fully half of our membership will be international very soon. The ASTMH Council is looking at ways to do more to bring the rich voices of our diverse membership to the Society. I hope that soon, you will be listening to the president's address given by someone from Bamako, or Beijing, or Lima.

I reminded us of the calls by earlier presidents for us individually and collectively to take up the tools of advocacy and diplomacy to improve global health. As a scientific and public health community, we bring a unique and neutral role to the policy and advocacy table. We do not have a hidden agenda. Our commitment to improved health is not rooted in a political party or in service to some political vision. Our political party is the human party.

Let us make it part of our mission and part of our routine to reach out nontraditional partners: think tanks like CSIS, members of Congress whether from red states or blue states, and even military rulers and armed militias; and find ways to promote peace and reconciliation and work together to achieve our vision: a peaceful world free of tropical diseases.

Let us put ourselves out of business.

